# High-Power-Density Energy-Harvesting Devices Based on the Anomalous Nernst Effect of Co/Pt Magnetic Multilayers

**DOI:** 10.1021/acsaem.2c02422

**Published:** 2022-09-09

**Authors:** Guillermo Lopez-Polin, Hugo Aramberri, Jorge Marques-Marchan, Benjamin I. Weintrub, Kirill I. Bolotin, Jorge I. Cerdá, Agustina Asenjo

**Affiliations:** † Instituto de Ciencia de Materiales de Madrid (ICMM-CSIC), 28049 Madrid, Spain; ‡ Materials Research and Technology Department, Luxembourg Institute of Science and Technology (LIST), L-4362 Esch-sur-Alzette, Luxembourg; § Department of Physics, Freie Universität Berlin, 14195 Berlin, Germany

**Keywords:** anomalous Nernst effect, energy harvesting, magnetic multilayers, perpendicular magnetic anisotropy, magnetic force microscopy

## Abstract

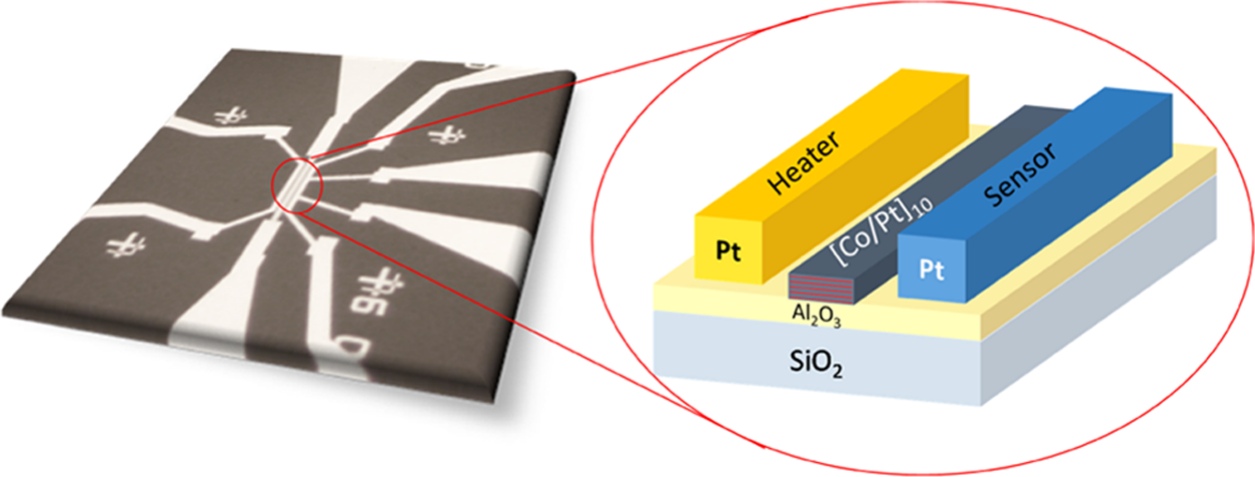

The anomalous Nernst effect (ANE) is a thermomagnetic phenomenon with potential applications in thermal energy harvesting. While many recent works studied the approaches to increase the ANE coefficient of materials, relatively little effort was devoted to increasing the power supplied by the effect. Here, we demonstrate a nanofabricated device with record power density generated by the ANE. To accomplish this, we fabricate micrometer-sized devices in which the thermal gradient is 3 orders of magnitude higher than conventional macroscopic devices. In addition, we use Co/Pt multilayers, a system characterized by a high ANE thermopower (∼1 μV/K), low electrical resistivity, and perpendicular magnetic anisotropy. These innovations allow us to obtain power densities of around 13 ± 2 W/cm^3^. We believe that this design may find uses in harvesting wasted energy, e.g., in electronic devices.

## Introduction

Thermoelectric generators, traditionally based on the Seebeck effect,^1^ are devices capable of harvesting energy from any source of wasted thermal energy, such as the heat emitted by the human body or by the Joule effect of different electronic devices. More than half of the considered waste heat streams arise at a low-temperature range, below 200 °C, of which microelectronics take an important share.^2^ This paper explores the opportunity of using thermoelectric effects for converting wasted heat from high-performance integrated circuits, such as microprocessors, into electric energy. During the last decade, thermoelectric generators based on the anomalous Nernst effect (ANE) have been proposed for their potential to achieve better efficiency.^3^ The ANE occurs in the presence of a thermal gradient perpendicular to a magnetic field, resulting in an electric field perpendicular to both.^3,4^ The effect is anomalous when it is produced by the intrinsic magnetization of the sample instead of an external magnetic field. The transverse nature of ANE (in contrast with the Seebeck effect) allows for lateral configurations with simpler design, higher performance, and much lower production cost. Recently, materials with increasing ANE coefficients and decreasing costs have been found.^5−7^ However, ANE generators still have some disadvantages compared to those based on the Seebeck effect. First, the maximum ANE thermopower measured so far^7,8^ is still 2 orders of magnitude lower than the maximum Seebeck coefficient found in an element.^9^ Moreover, while the Seebeck effect is proportional to the gradient of temperature, the ANE depends also on the component of the magnetization perpendicular to the thermal gradient (*E⃗*_ANE_ ∝ *m⃗* × ∇⃗*T*). Therefore, having magnetization in a well-defined direction becomes crucial for the enhancement of this effect. Magnetic multilayers showing high perpendicular magnetic anisotropy (PMA) have been studied for a long time because of their critical role in the development of magnetic recording,^10^ high-density nonvolatile memories,^11^ interface-induced phenomena,^12^ emergent spin-electronic technology,^13^ and the discovery of the magnetic skyrmions,^14^ but materials with PMA are also very advantageous for maximizing the ANE voltage owing to their high remanent magnetization along the out-of-plane direction.^15−17^

The ANE can be viewed as the thermal analogue of the anomalous Hall effect (AHE) in magnetic materials.^7^ The AHE causes the appearance of a voltage difference across a ferromagnetic electrical conductor perpendicular to an electric current flowing through the material. In the ANE, the driving current is caused by the Seebeck effect (i.e., parallel to the thermal gradient), resulting in an electric field transverse to the thermal gradient. The AHE is a phenomenon where the transport properties of the spin-polarized electrons are governed by the spin–orbit coupling (SOC), which couples the orbital and spin degrees of freedom of the electron.^18^ Consequently, SOC plays a crucial role in both AHE and ANE.^7^ Both effects are connected to the electrical conductivity through the Mott relations.^19^ Most of the materials with high ANE also show relatively large remanent magnetization. In the last few years, however, it was shown that a substantial contribution to the ANE originates from the Berry curvature close to the Fermi level,^20^ and hence is strongly affected by topological band crossings (like Weyl nodes) that act as sources or sinks of the Berry curvature (and are themselves affected by the magnetization). Following this realization, some materials with large ANE and low magnetization have been reported.^21^ This contribution is often called “intrinsic”, as opposed to other so-called extrinsic mechanisms like magnon drag,^7,22^ phonon drag,^23^ or skew forces due to large spin–orbit coupling in ferromagnetic metals,^24,25^ which have also been proposed to yield large contributions to the ANE.

Most of the works published about the ANE coefficient of different materials focus on the ANE voltage, but no attention has been paid to the maximum power supplied by the ANE effect. Power supplied by the ANE will play a major role in determining the feasibility of the material for energy-harvesting applications. In this work, we study the ANE response of Co/Pt multilayers with high PMA, in which the magnetization of the system is well oriented perpendicular to the layers. In addition, the miniaturization of the heat source produces the thermal decay to occur in a reduced space, thus enhancing the thermal gradient (in K/m). Therefore, from miniaturized devices, we expect to obtain higher voltages and current densities. Accordingly, we evaluate the power supplied by Co/Pt devices with micrometer lateral dimensions, obtaining record power densities supplied by our devices. Our data suggest that thermoelectric devices based on the Nernst effect can be very effective to harvest wasted thermal energy from nano- or microscopic heating sources like microelectronic devices.

## Experimental Section

We first explore the ANE response in macroscopic devices based on Co/Pt multilayer samples. To evaluate the influence of the Co and Pt thicknesses, we fabricate several samples on SiN*_x_*/Si substrates with different Co and Pt thicknesses and a fixed number of repetitions (namely, 10 repetitions). The ANE response of these multilayers is evaluated using the setup for characterizing the ANE of macroscopic devices described in Supporting Information Section 1(a). We use the configuration with the in-plane temperature gradient and the out-of-plane magnetic fields to avoid contributions from the spin Seebeck effect.^17^ In addition, this setup allows us to determine the temperature gradient more precisely to correctly characterize the ANE thermopower. The maximum ANE response in saturation was found for the multilayers with 0.5 nm Co and 1.5 nm Pt, which show high PMA as deduced from the magnetic hysteresis loops (SI Section 1(a)) measured by a vibrating sample magnetometer (VSM). Hereafter, we focus on the characterization of the devices based on these [Co_0.5nm_Pt_1.5nm_]_10_ multilayers (TEM characterization in SI Section 1(b)).

**Figure 1:**
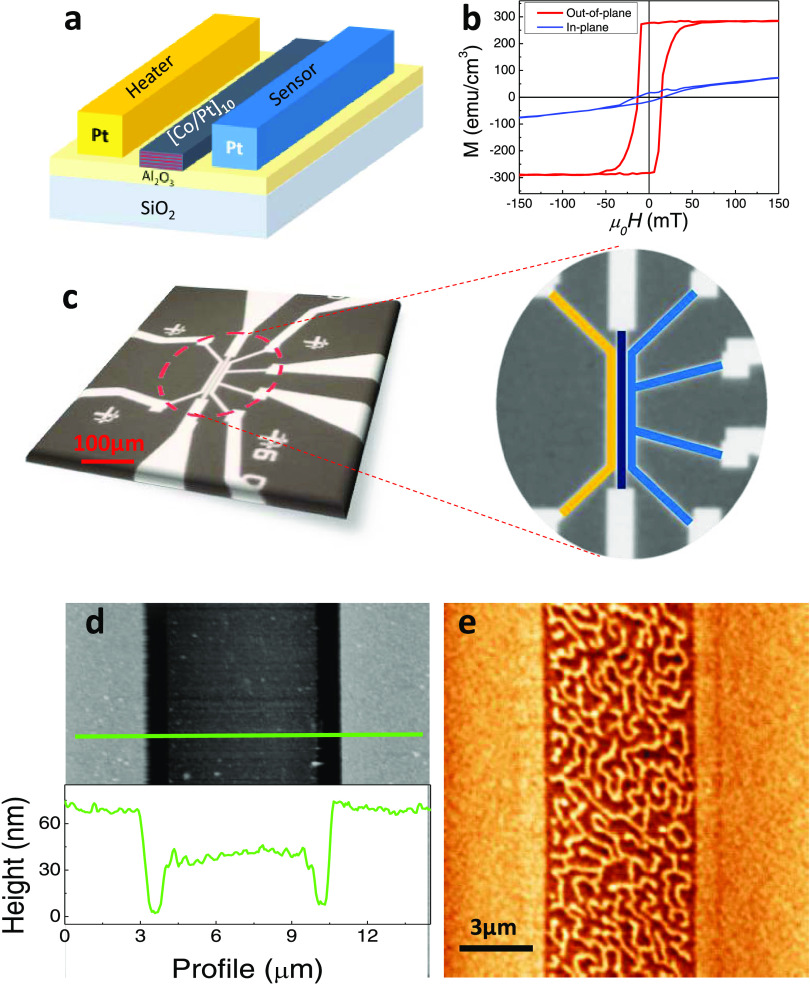
(a) Cartoon showing a schematic of the device. (b) In-plane (blue) and out-of-plane (red) hysteresis loops. (c) Optical image of the device and the zoomed view with colored regions corresponding to the stripe (dark blue), the heater (yellow), and the sensor (light blue). (d) Atomic force microscopy (AFM) topography of the heater, Co/Pt multilayer, and sensor. The green line corresponds to the line of the profile shown below. (e) Magnetic force microscopy (MFM) image of the magnetic stripe in a demagnetized state.

To achieve higher thermal gradients and optimize the voltage per area, we fabricate microscopic devices as illustrated in Figure 1a (more details in SI Section 1(c)). The 100 × 5 μm^2^ stripe at the center (dark blue colored in the zoomed view of Figure 1c) is the Co/Pt multilayer. The rest of the patterns were made of Pt: the bar on the left (orange) acts as a heater, and the bar on the right (light blue) works as a four-terminal thermal sensor. The high PMA of the selected multilayer [Co_0.5nm_Pt_1.5nm_]_10_ is revealed from the in-plane and out-of-plane hysteresis loops represented in Figure 1b, showing high out-of-plane remanent magnetization (close to the saturation value) and low in-plane remanence. In addition, hysteresis loops show a remanence of the magnetization of ∼100% along the perpendicular direction with no applied field, in contrast with other previously reported materials with a high ANE coefficient but very low remanence.^5,6,26^ The hysteresis loops were measured by VSM on 5 × 5 mm^2^ samples grown in parallel with the multilayer structure of the microscopic devices. In the following, all of the reported experiments are performed on the microfabricated devices. We measure the magnetic state of the multilayer by magnetic force microscopy (MFM) using an amplitude modulation mode and a low magnetic moment tip (SI Section 6). Figure 1d shows the topography, and the profile shows the geometry of the device: the thickness of the multilayers, the heater, and the thermometer, as well as the distance between them. Figure 1e shows the corresponding MFM image obtained in a demagnetized state at a retrace distance of 30 nm. The phase-locked loop feedback is activated to keep the phase constant, and thus the magnetic signal is in the frequency shift channel.^27^ The variation of the resonance frequency at this distance mostly depends on long-range conservative forces (i.e., magnetic and electrostatic forces). The MFM images in this state exhibit stripe domains. From these images, we can extract the normalized remanent magnetization of the sample in the perpendicular direction, *M*_RZ_, from the following equation^28^
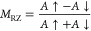
1where *A*↑ is the area of the domains pointing up and *A*↓ is the area of the domains pointing down. We assume a constant magnetization across the thickness of the sample. To characterize the magnetization in situ and relate the ANE response with the domain structure of the multilayer, we measure the ANE voltage while simultaneously obtaining MFM images and varying the magnetization of the sample with a perpendicular field created by an electromagnet below the sample.^29^

A key issue to accurately evaluate the ANE thermopower and coefficient of the devices is to extract the temperature gradient from the average temperature of the Pt sensor, which is the magnitude measurable in our experiments. We carry out COMSOL Multiphysics simulations^30^ to extract the temperature gradient in the Co/Pt structure from the average temperature of the platinum sensor. A more detailed description of the finite-element simulations and the method for determining the temperature gradient can be found in SI Section 2. We find that the mean thermal gradient across the multilayer structure has a linear dependence with the average temperature of the sensor of about 0.08 K/μm per K measured in the resistance. This value will be used to calculate the thermal gradient in the Co/Pt element based on the experimental values of the Pt sensor. The relationship between the resistance of the Pt sensor and the temperature is determined experimentally. A linear dependence of 2700 ppm/K is obtained, much lower than the value for bulk Pt^31^ (SI Section 3).

## Results and Discussion

**Figure 2:**
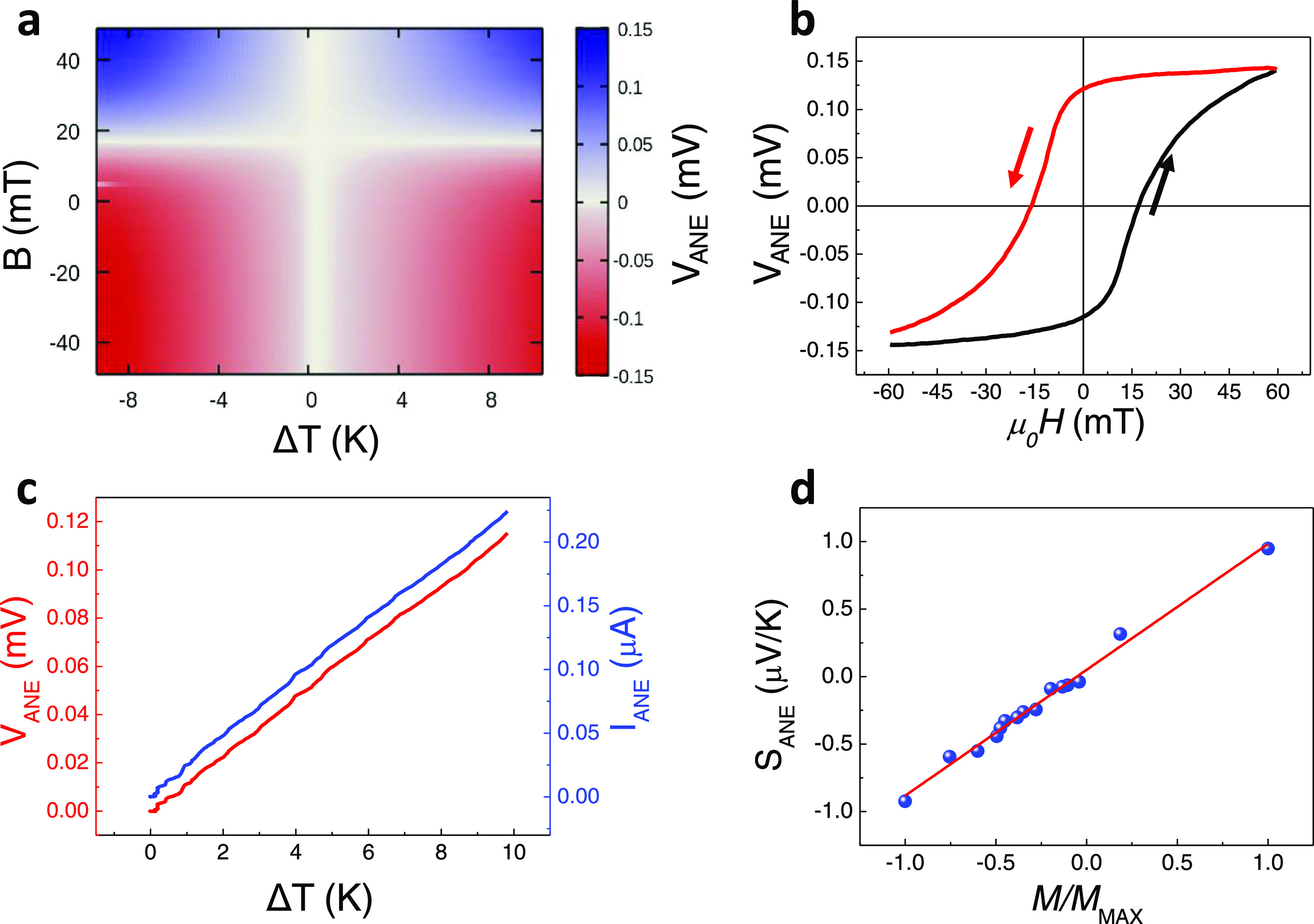
(a) Map of the Nernst voltage as a function of the thermal gradient (*X*-axis) and the applied magnetic field (*Y*-axis). Note that the negative sign of the thermal gradient only indicates the direction of the current passing through the heater. (b) Hysteresis loop of the Nernst voltage vs the applied field. (c) Nernst voltage (red) and the generated current (blue) as a function of the thermal gradient. (d) ANE thermopower as a function of the magnetization of the sample.

The characterization of the device has been performed using different strategies: a complete map of the ANE voltage vs temperature and magnetic field and individual curves of ANE voltage either vs temperature (in remanence) or vs magnetic field (at a fixed temperature). To experimentally control the thermal gradient variation, we sweep the current passing through the heater. Similarly, the in situ out-of-plane magnetic field can be modified continuously.^29^Figure 2a shows a map of the ANE voltage (*V*_ANE_) as a function of the thermal gradient across the multilayer (varying the current from negative to positive values along the fast scan, *X*-axis) and the out-of-plane magnetic field (slow scan, *Y*-axis). First, we measure *V*_ANE_ vs temperature gradient at the maximum negative field (−45 mT) that we can maintain in the system for long periods of time without overheating the coil (bottom of the map in Figure 2a). Then, we apply increasing magnetic fields from negative to positive values while *V*_ANE_ vs temperature curves are measured at each field. As the heater increases the temperature with the current, regardless of the direction, the Nernst voltage vs the current presents a V shape. This allows us to discard possible current leaks from the heater or the sensor to the structure. Figure 2b shows the hysteresis loop of *V*_ANE_ at a constant thermal gradient of ∼2.5 K/μm, sweeping the field from −60 to 60 mT (which is close to the saturation field with >90% alignment). This data is in good agreement with Figure 2a since similar behavior can be found by plotting a vertical profile through the three-dimensional (3D) map. Note that the hysteretic behavior is similar to the magnetization vs field plot, evidencing the thermomagnetic origin of the effect.

Besides, the in situ *V*_ANE_ signal is measured as a function of the temperature gradient in the remanence state. The *V*_ANE_ exhibits a linear dependence on temperature (Figure 2c, additional information is given in SI Sections 4 and 5). Similar information can be obtained from the horizontal profiles in Figure 2a, which corresponds to a *V*_ANE_ curve vs temperature under an applied magnetic field. The MFM images obtained in remanence, after applying a saturating out-of-plane magnetic field ex situ, present a uniform contrast, which corresponds to a remanence of ∼100%. (SI Section 6). Finally, we repeat these curves (ANE response vs the current applied to the heater) for the remanent states reached after applying different magnetic fields. The magnetization of the sample is evaluated using the MFM images. The ANE thermopower (*S_yx_* = *S*_ANE_) of the sample was extracted by multiplying the slope of each curve (total voltage divided by the temperature difference across the whole multilayer in μV/K) by the geometrical factor of the magnetic stripe (length divided by width). Figure 2d shows the *S*_ANE_ as a function of the remanent magnetization of the sample. As expected, the *S*_ANE_ exhibits a linear trend with the magnetization and reaches the same absolute value at *M*/*M*_max_ = 1 and at *M*/*M*_max_ = −1. When the sample is saturated, we obtain a value of 0.95 μV/K, very similar to the ANE obtained on the macroscopic device corresponding to the same multilayered sample (Co_0.5_Pt_1.5_). From the slope, we can extract the Nernst coefficient, which is defined by
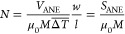
2where μ_0_ is the vacuum permeability, *M* is the maximum magnetization of the structure, Δ*T̅* is the average thermal gradient, *w* is the width, and *l* is the length of the multilayer bar. The Nernst coefficient obtained for this multilayer is *N* = 2.3 μV/(T·K).

To make an ANE device with high energy-harvesting potential, it is also crucial to evaluate the maximum power that the device can provide. We measured the current supplied by the multilayer as a function of the thermal gradient by grounding one of the sides of the device through a current-to-voltage converter while applying a thermal gradient. Note that the larger the thermal gradient, the higher the voltage (and current) obtained (see Figure 2c). However, the maximum performance of the device is limited by the maximum temperature admissible until the magnetic moment starts to decrease due to thermal fluctuations (SI Section 4). In this case, the maximum voltage and current are ∼0.3 mV and ∼0.6 μA, respectively, with a total thermal gradient of ∼4 K/μm. For comparison, the maximum gradient of temperatures achieved on the macroscopic devices is around 0.003 K/μm. It is important to consider that the measurement of the current was performed with two terminals. Thus, the current is not only dictated by the resistance of the structure but also by the contact resistance. In fact, the resistance of the multilayer measured in two contacts was about 500 Ω, which is just the voltage divided by the obtained current. Therefore, the measured value (0.6 μA) is just a lower limit of the maximum current that can be supplied by the device but it could be higher if the contact resistance is reduced. The maximum power supplied by the device is *P* = *IV*, which gives approximately 180 pW. The power scales linearly with every dimension of the structure: voltage scales linearly with the length of the device while the current remains constant, and the current increases proportionally to the width and thickness of the multilayer with no variation of the voltage. At the largest temperature gradient, the maximum voltage per length obtained in remanence is 30 mV/cm, and the maximum current per section area is 480 A/cm^2^. From three different samples, we obtained a maximum power density of 13 ± 2 W/cm^3^. Note that Seebeck generators usually give power densities in the order of tens of mW/cm^3^.^32^

**Figure 3:**
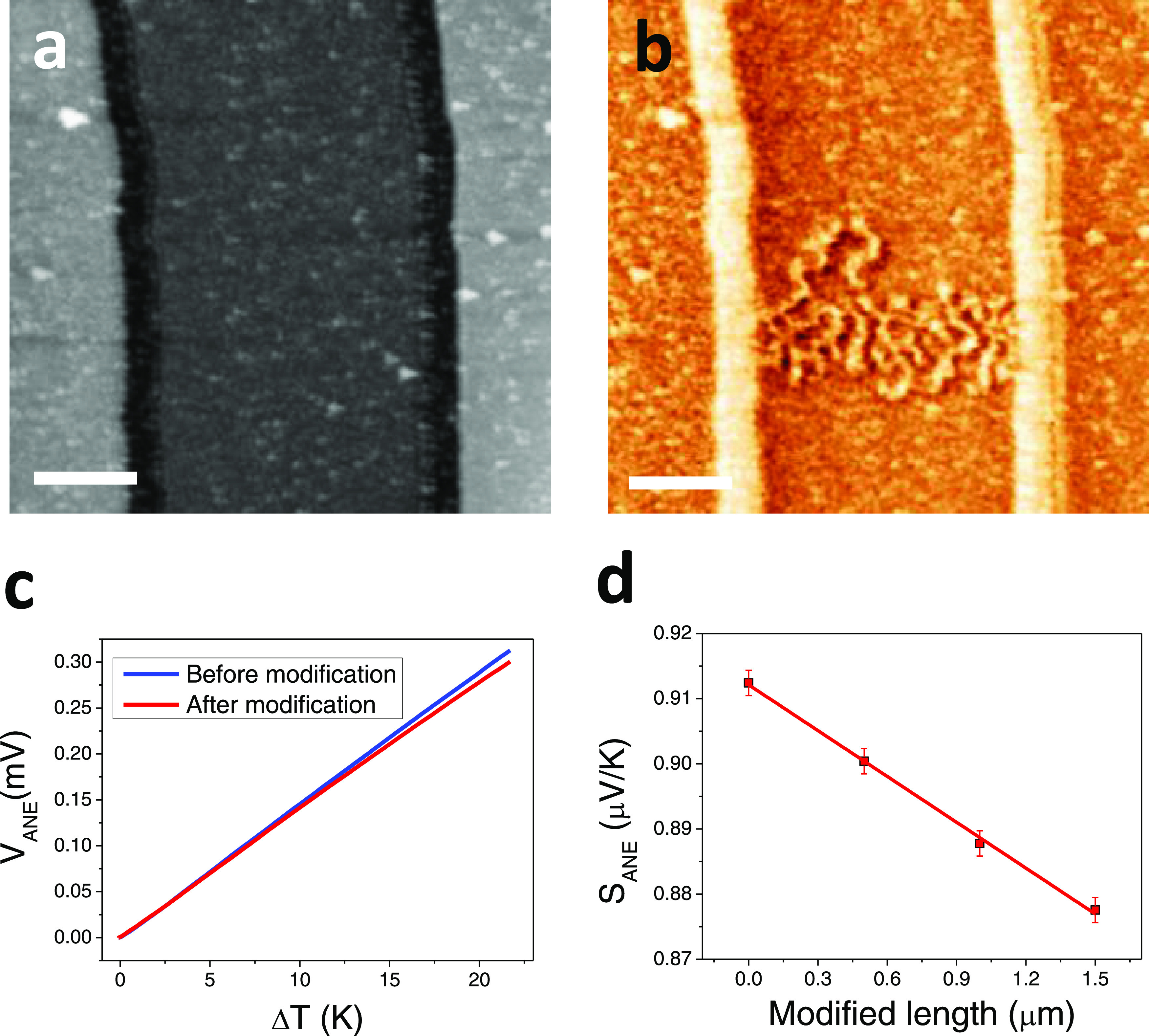
(a) Topography and (b) MFM image of the device after modification, (c) ANE voltage (before and after modification) vs temperature, and (d) variation of the ANE thermopower with the length (proportional to the volume and the total magnetization) of the modified region.

The high ANE voltages obtained from the devices due to the high thermal gradients achieved and the high Nernst coefficient of the multilayers make it possible to detect small variations of the magnetization of the device. To quantify the limit of the sensitivity of the device, we induced slight variations in the total magnetization of the sample. With the tip stray field, antiparallel to the sample magnetization, it is possible to write domains inducing local changes in the magnetic state^15^ just by approaching the MFM tip to certain regions of the sample. With just one sweep, we inverted around 40% of the scanned area, around 0.25 × 5 μm^2^ (Figure 3a,b). This slight variation of the total magnetization of the sample, around ∼0.1%, can be detected by comparing the *V*_ANE_ before and after the writing process, as shown in Figure 3c. The expected linear decay of the |*S*_ANE_| vs the magnetic moment of the sample (proportional to the volume of the sample pointing in the down direction) is shown in Figure 3d. The sensitivity of the device is enough to detect human heat in ambient conditions (see SI Section 9).

The ANE of other materials with PMA has also been measured by other groups.^15−17,33^ While some of them present an ANE thermopower lower than that reported here (e.g., L_10_-ordered epitaxial FePt thin films^16^ and multilayers of MgO/Co_2_MnGa/Pd^33^), others portray a Nernst thermopower almost twice as large (e.g., IrMn/CoFeB/MgO^17^). However, the resistivity of IrMn/CoFeB/MgO should be very high because most of the layers show very poor conductivity: MgO is an insulator and CoFeB presents high resistivity.^34^ Therefore, the maximum power provided by these multilayers should be much lower than that reported in this work. In previous work on MnBi,^7^ a large ANE was attributed to an extrinsic magnon-drag effect, which is most likely to occur in ferromagnetic samples with large SOC, as the one discussed here. Besides, the ANE thermopower of Fe/Pt multilayers was reported to be ∼1 μV/K,^35^ very similar to the value measured in this work. The advantage of our system is its high PMA, which simplifies the design of a device, allowing it to easily align the direction of the thermal gradient perpendicular to the magnetization to obtain the maximum ANE voltage. The high ANE measured on Fe/Pt multilayers was attributed to an unconventional interface-induced enhancement thermoelectric conversion in the Fe films, not to the proximity effect-induced magnetism on the Pt by the Fe layers. Also, UCo_0.8_Ru_0.2_Al was reported to have a colossal ANE of 23 μV/K and a large ANE conductivity of 15 A/(m·K) at ∼40 K.^8^ However, above 60 K, the material is not ferromagnetic and does not present ANE. The authors claim that to enhance the Berry curvature contribution to the ANE, in addition to a large spin–orbit coupling (SOC) and a strong electronic correlation, a kagome lattice structure can be of help. Theoretical calculations show that there are several Weyl nodes close to the Fermi level on this material. In another work on YMnBi_2_,^24^ the large ANE measured in the samples could not be explained from first-principles-based calculations (which typically only account for the intrinsic contribution) of the Nernst thermopower obtained with the Mott relation. Scattering (unavoidable in real samples, particularly in those with large SOC) induces skew forces and side jump on the spin-polarized charge carriers.^24^ Moreover, the net ANE can have contributions of all of the mentioned origins (including the Berry curvature). It is even possible that none of them is predominant. However, testing all of these possibilities falls beyond the reach of the present work.

**Figure 4:**
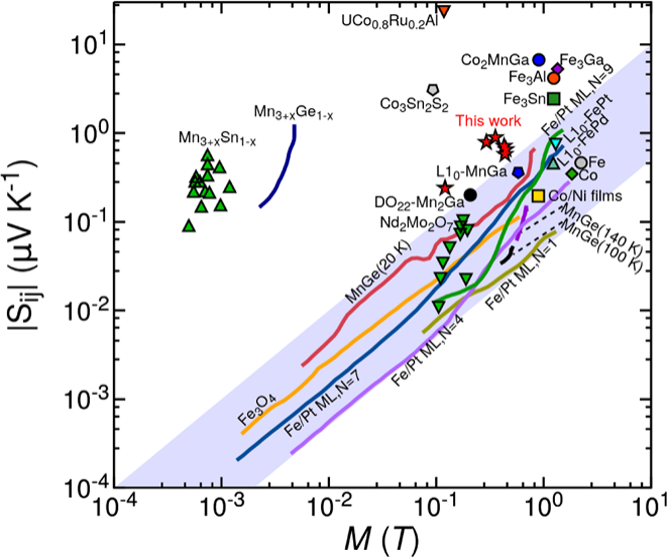
Anomalous Nernst thermopower as a function of spontaneous magnetization for several materials reported in the literature. We include data for Fe_3_O_4_ (orange line);^19^ Mn_3+_*_x_*Sn_1–*x*_ (green upward triangles);^21^ Mn_3+*x*_Ge_1–*x*_ (dark blue line);^36^ Co_2_MnGa (blue circle);^26^ Co_3_Sn_2_S_2_ (gray pentagon);^37^ UCo_0.8_Ru_0.2_Al (orange downward triangle);^8^ Fe_3_Ga (purple diamond);^6^ Fe_3_Al (orange circle);^6^ Fe_3_Sn (green square);^38^ Fe/Pt multilayers with *N* interfaces^35^ with *N* = 1 (ocher line), *N* = 4 (light purple line), *N* = 7 (blue line), and *N* = 9 (green line); MnGe^39^ at *T* = 20 K (red line), *T* = 100 K (black lines), and *T* = 140 K (dark purple line); Nd_2_Mo_2_O_7_ (green downward triangles), Fe (gray circle), and Co (green diamond);^40^ and L_10_-FePd (turquoise upward triangle), L_10_-FePt (cyan downward triangle), L_10_-MnGa (blue pentagon), D_022_-Mn_2_Ga (black circle),^41^ Co/Ni films (yellow square),^41^ and the Co/Pt multilayers studied in this work (red stars). The shaded region indicates the linear relation for conventional ferromagnetic metals.

To put into perspective our results, Figure 4 shows the values of anomalous Nernst thermopowers for several works in the literature. The shaded region is that of |*S*_*ij*_| = |*Q*_s_|μ_0_*M* with |*Q*_s_| ranging from 0.05 to 0.1 μV/(K·T), where most of the reported ANE thermopowers lie. The Co/Pt multilayers studied here fall outside this region, like Mn_3+*x*_Sn_1–*x*_ or Co_2_MnGa, which have been shown to display a large intrinsic ANE.

**Figure 5:**
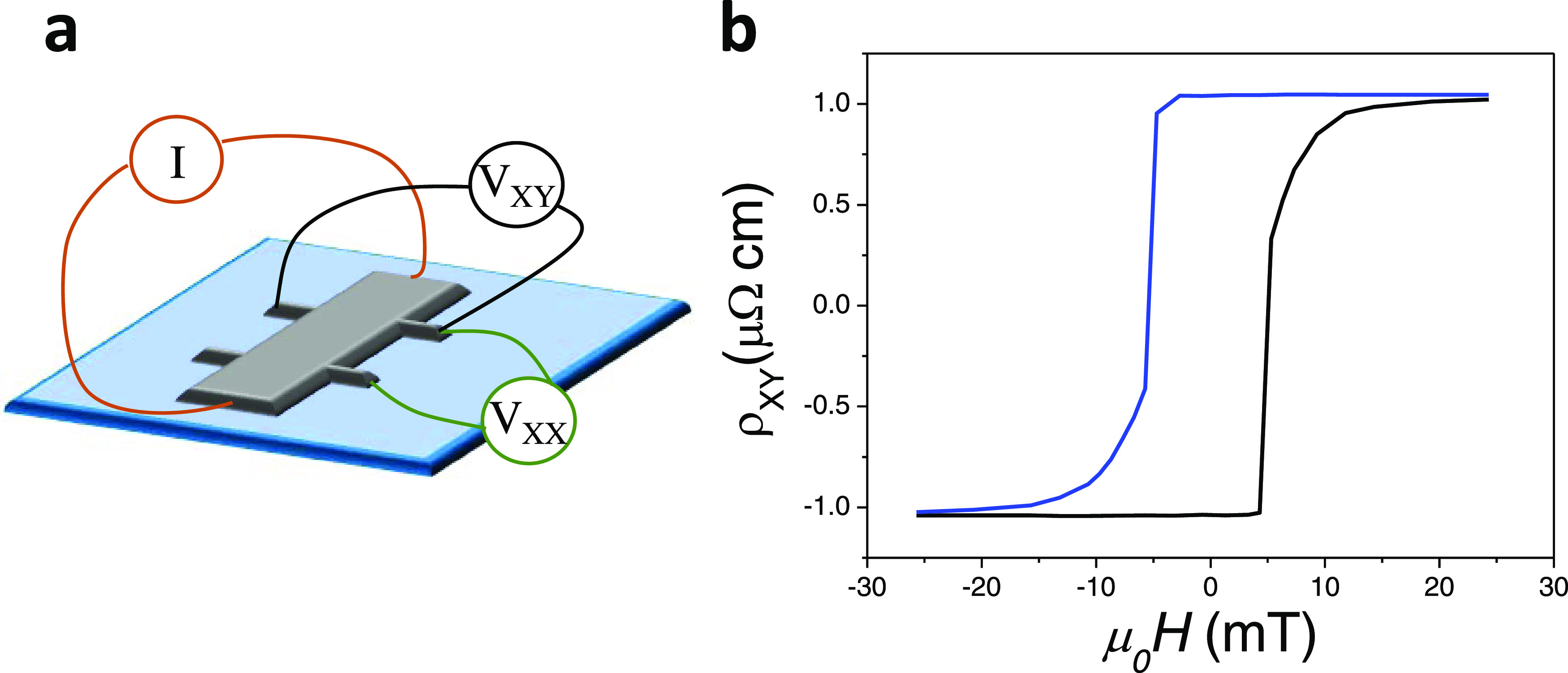
(a) Cartoon showing the setup used to measure the AHE of the Co/Pt multilayers. (b) AHE resistivity vs applied field of the Co/Pt multilayer. The measurements are performed at room temperature (RT) with the magnetic field applied perpendicular to the sample. The black line is measured from negative to positive fields and the blue line is measured from positive to negative fields.

To elucidate the origin of the large ANE in our Co/Pt multilayers, we performed AHE measurements^42^ of the [Co_0.5nm_Pt_1.5nm_]_10_ multilayers and carried out first-principles calculations (details in SI Section 7). To this end, we grow a Hall bar of Co/Pt. We measured the longitudinal (ρ*_xx_*) and transversal (ρ*_xy_*) resistivity at room temperature (300 K) of the multilayer as a function of an external field (Figure 5 and SI Section 9). We observed that the ρ*_xy_* vs field (Figure 5) has a similar shape to the magnetization vs field curve. The AHE resistivity saturates at ρ*_xy_* ∼ 1 μΩ·cm, and no increase is detectable for higher fields, which means that the anomalous contribution to the AHE is much larger than that of the ordinary Hall effect. On the other hand, the longitudinal resistivity (SI Section 8) shows no variations with the field and remains constant at ρ*_xx_* ∼ 50 μΩ·cm. This data is in good agreement with other works determining the longitudinal and Hall resistivity of Co/Pt multilayers.^43,44^ While the longitudinal resistivity falls at the limit between the dirty (or bad metal) regime and the intrinsic regime, the very low Hall resistivity falls well beyond the universal scaling law for anomalous transport for bulk systems.^42^ Previous works^43,44^ on the origin of the anomalous Hall transport on these multilayers point to interface scattering as the most important contribution to the AHE, which also modifies the relationship between ρ*_xx_* and ρ*_xy_*, and explains the deviation of the multilayers from the universal scaling law of the transversal and longitudinal conductivity for bulk materials.

From first-principles calculations, we obtain the (intrinsic) thermoelectric conductivity tensor (α_*ij*_) of Co/Pt heterostructures (see SI Section 7). We explore different thicknesses of the Pt layer (from 2 to 5 monolayers), while the Co layer is fixed (to two monolayers). The results, shown in Figure S7, indicate that the intrinsic ANE would change sign with the addition of each Pt monolayer. From the transport measurements, we can estimate the experimental thermoelectric conductivity of the Co_0.5nm_Pt_1.5nm_ multilayer through , where ρ_*xx*_ is the longitudinal resistivity, ρ_*xy*_ is the Hall resistivity, and *S*_xx_ is the Seebeck coefficient.^45^ We obtain α_*xy*_ = 2.5 A/(K·m), relatively far from the computed value for the 2Co/5Pt heterostructure (1.2 A/(K·m)), whose layer thicknesses are the closest to the experimental ones among those calculated. More importantly, the ANE contribution to the experimental thermoelectric conductivity dominates over that of the AHE (the former is more than 2 times larger than the latter) and can be expected to dominate at other Pt thicknesses (note that the ρ_*xy*_ has been shown to vary only modestly with Pt thickness^46^). Since the measured ANE thermopowers vary only mildly with Pt thickness, we anticipate that the experimental thermoelectric conductivities will also vary only slightly with Pt thicknesses. This is at odds with the computed (intrinsic) thermoelectric conductivities, which depend very strongly on the thickness (note that in other systems, the first-principles-based calculations of the intrinsic ANE also fail to fully explain the origin of large Nernst thermopower^24^). Hence, this indicates again that the origin of the main contribution to the ANE of Co/Pt multilayers is not intrinsic. Our simulations thus point to extrinsic effects as the main contribution to the large ANE in this system.

## Conclusions

Our results show that Co/Pt multilayers present a high ANE coefficient and thermopower. Materials with large PMA are ideal candidates for the development of ANE devices due to their well-defined magnetization direction that allows them to optimize and improve their performance as energy harvesters. Moreover, we propose to enhance the power densities of the devices by miniaturization to the micro- or nanoscale. We obtained power densities of 13 ± 2 W/cm^3^. Thus, usable powers could be obtained by multilayers thicker than that presented here and by parallelizing several devices. In contrast to the insulators that present high ANE thermopower, the advantage of multilayered metallic systems is their low electrical resistance that allows us to achieve high current densities. In addition, the technical growth requirements make this system affordable and easily achievable.

To elucidate the origin of the high ANE thermopower on the multilayers, we present theoretical simulations and AHE measurements. Theoretical simulations suggest that the origin of the high ANE is not intrinsic. The longitudinal and AHE resistivity is similar to that reported in previous works, where interface scattering is found to be the main contribution to the AHE in similar multilayers with strong PMA. Therefore, we attribute the high ANE of Co/Pt multilayers also to the interface scattering.

In summary, the ANE exhibited by the ferromagnet/heavy metal (FM/HM) multilayers can be a novel approach to energy sustainability due to its potential to produce electric harvesting devices with large power densities. One additional benefit of this system is its ability to develop functional devices on a variety of substrates, including flexible ones. This could allow us to fabricate wearable thermoelectric generators, which would be useful to feed wearable electronic devices for medical applications or related to the Internet of Things.

## Supporting Information Available

Device fabrication; atomic structure of the multilayers as measured by TEM; COMSOL simulations; calibration of the dependence of the resistivity of the platinum bar with temperature; MFM images of the microscopic devices; maximum temperature gradient and ANE voltage in remanence; *V*_ANE_ vs Δ*T* curves from experimental data; first-principles calculations; anomalous Hall effect measurements; and sensitivity of macroscopic devices (PDF)

## References

[ref1] Jaziri N.; Boughamoura A.; Müller J.; Mezghani B.; Tounsi F.; Ismail M. A comprehensive review of Thermoelectric Generators: Technologies and common applications. Energy Rep. 2020, 6, 264–287. 10.1016/j.egyr.2019.12.011.

[ref2] Xu Z.; Wang R.; Yang C. Perspectives for low-temperature waste heat recovery. Energy 2019, 176, 1037–1043. 10.1016/j.energy.2019.04.001.

[ref3] Mizuguchi M.; Nakatsuji S. Energy-harvesting materials based on the anomalous Nernst effect. Sci. Technol. Adv. Mater. 2019, 20, 262–275. 10.1080/14686996.2019.1585143. 30956732 PMC6442159

[ref4] Bauer G. E. W.; Saitoh E.; Van Wees B. J. Spin caloritronics. Nat. Mater. 2012, 11, 391–399. 10.1038/nmat3301. 22522639

[ref5] Sakai A.; Mizuta Y. P.; Nugroho A. A.; Sihombing R.; Koretsune T.; Suzuki M.-T.; Takemori N.; Ishii R.; Nishio-Hamane D.; Arita R.; et al. Giant anomalous Nernst effect and quantum-critical scaling in a ferromagnetic semimetal. Nat. Phys. 2018, 14, 1119–1124. 10.1038/s41567-018-0225-6.

[ref6] Sakai A.; Minami S.; Koretsune T.; Chen T.; Higo T.; Wang Y.; Nomoto T.; Hirayama M.; Miwa S.; Nishio-Hamane D.; et al. Iron-based binary ferromagnets for transverse thermoelectric conversion. Nature 2020, 581, 53–57. 10.1038/s41586-020-2230-z. 32376952

[ref7] He B.; Şahin C.; Boona S. R.; Sales B. C.; Pan Y.; Felser C.; Flatté M. E.; Heremans J. P. Large magnon-induced anomalous Nernst conductivity in single-crystal MnBi. Joule 2021, 5, 3057–3067. 10.1016/j.joule.2021.08.007. 34841198 PMC8604385

[ref8] Asaba T.; Ivanov V.; Thomas S.; Savrasov S.; Thompson J.; Bauer E.; Ronning F. Colossal anomalous Nernst effect in a correlated noncentrosymmetric kagome ferromagnet. Sci. Adv. 2021, 7, eabf1467 10.1126/sciadv.abf1467. 33771869 PMC7997519

[ref9] Henkels H. W. Thermoelectric power and mobility of carriers in selenium. Phys. Rev. 1950, 77, 734 10.1103/PhysRev.77.734.

[ref10] Moser A.; Takano K.; Margulies D. T.; Albrecht M.; Sonobe Y.; Ikeda Y.; Sun S.; Fullerton E. E. Magnetic recording: advancing into the future. J. Phys. D: Appl. Phys. 2002, 35, R157 10.1088/0022-3727/35/19/201.

[ref11] Ikeda S.; Miura K.; Yamamoto H.; Mizunuma K.; Gan H.; Endo M.; Kanai S.; Hayakawa J.; Matsukura F.; Ohno H. A perpendicular-anisotropy CoFeB–MgO magnetic tunnel junction. Nat. Mater. 2010, 9, 721–724. 10.1038/nmat2804. 20622862

[ref12] Hellman F.; Hoffmann A.; Tserkovnyak Y.; Beach G. S. D.; Fullerton E. E.; Leighton C.; MacDonald A. H.; Ralph D. C.; Arena D. A.; Dürr H. A.; et al. Interface-induced phenomena in magnetism. Rev. Mod. Phys. 2017, 89, 025006 10.1103/RevModPhys.89.025006. 28890576 PMC5587142

[ref13] Dieny B.; Chshiev M. Perpendicular magnetic anisotropy at transition metal/oxide interfaces and applications. Rev. Mod. Phys. 2017, 89, 025008 10.1103/RevModPhys.89.025008.

[ref14] Fert A.; Reyren N.; Cros V. Magnetic skyrmions: advances in physics and potential applications. Nat. Rev. Mater. 2017, 2, 17031 10.1038/natrevmats.2017.31.

[ref15] Scarioni A. F.; Barton C.; Corte-León H.; Sievers S.; Hu X.; Ajejas F.; Legrand W.; Reyren N.; Cros V.; Kazakova O.; Schumacher H. W. Thermoelectric signature of individual skyrmions. Phys. Rev. Lett. 2021, 126, 077202 10.1103/PhysRevLett.126.077202. 33666484

[ref16] Mizuguchi M.; Ohata S.; Uchida K.-i.; Saitoh E.; Takanashi K. Anomalous Nernst effect in an L10-ordered epitaxial FePt thin film. Appl. Phys. Express 2012, 5, 093002 10.1143/APEX.5.093002.

[ref17] Tu S.; Hu J.; Yu G.; Yu H.; Liu C.; Heimbach F.; Wang X.; Zhang J.; Zhang Y.; Hamzić A.; et al. Anomalous Nernst effect in Ir_22_Mn_78_/Co_20_Fe_60_B_20_/MgO layers with perpendicular magnetic anisotropy. Appl. Phys. Lett. 2017, 111, 222401 10.1063/1.4996399.

[ref18] Bhowal S.; Satpathy S. Electric field tuning of the anomalous Hall effect at oxide interfaces. npj Comput. Mater. 2019, 5, 61 10.1038/s41524-019-0198-8.

[ref19] Ramos R.; Aguirre M.; Anadón A.; Blasco J.; Lucas I.; Uchida K.; Algarabel P.; Morellón L.; Saitoh E.; Ibarra M. Anomalous Nernst effect of Fe_3_O_4_ single crystal. Phys. Rev. B 2014, 90, 054422 10.1103/PhysRevB.90.054422.

[ref20] Xiao D.; Yao Y.; Fang Z.; Niu Q. Berry-phase effect in anomalous thermoelectric transport. Phys. Rev. Lett. 2006, 97, 026603 10.1103/PhysRevLett.97.026603. 16907470

[ref21] Ikhlas M.; Tomita T.; Koretsune T.; Suzuki M.-T.; Nishio-Hamane D.; Arita R.; Otani Y.; Nakatsuji S. Large anomalous Nernst effect at room temperature in a chiral antiferromagnet. Nat. Phys. 2017, 13, 1085–1090. 10.1038/nphys4181.

[ref22] Blatt F. J.; Flood D.; Rowe V.; Schroeder P.; Cox J. Magnon-drag thermopower in iron. Phys. Rev. Lett. 1967, 18, 395 10.1103/PhysRevLett.18.395.

[ref23] Ziman J. M. Electrons and Phonons: the Theory of Transport Phenomena in Solids; Oxford university press, 2001.

[ref24] Pan Y.; Le C.; He B.; Watzman S. J.; Yao M.; Gooth J.; Heremans J. P.; Sun Y.; Felser C. Giant anomalous Nernst signal in the antiferromagnet YbMnBi_2_. Nat. Mater. 2021, 203–209. 10.1038/s41563-021-01149-2. 34811495 PMC8810386

[ref25] Watzman S. J.; Duine R. A.; Tserkovnyak Y.; Boona S. R.; Jin H.; Prakash A.; Zheng Y.; Heremans J. P. Magnon-drag thermopower and Nernst coefficient in Fe, Co, and Ni. Phys. Rev. B 2016, 94, 144407 10.1103/PhysRevB.94.144407.

[ref26] Guin S. N.; Manna K.; Noky J.; Watzman S. J.; Fu C.; Kumar N.; Schnelle W.; Shekhar C.; Sun Y.; Gooth J.; Felser C. Anomalous Nernst effect beyond the magnetization scaling relation in the ferromagnetic Heusler compound Co_2_MnGa. NPG Asia Mater. 2019, 11, 16 10.1038/s41427-019-0116-z.

[ref27] Kazakova O.; Puttock R.; Barton C.; Corte-León H.; Jaafar M.; Neu V.; Asenjo A. Frontiers of magnetic force microscopy. J. Appl. Phys. 2019, 125, 060901 10.1063/1.5050712.

[ref28] Escrig J.; Altbir D.; Jaafar M.; Navas D.; Asenjo A.; Vázquez M. Remanence of Ni nanowire arrays: Influence of size and labyrinth magnetic structure. Phys. Rev. B 2007, 75, 184429 10.1103/PhysRevB.75.184429.

[ref29] Jaafar M.; Gómez-Herrero J.; Gil A.; Ares P.; Vázquez M.; Asenjo A. Variable-field magnetic force microscopy. Ultramicroscopy 2009, 109, 693–699. 10.1016/j.ultramic.2009.01.007. 19250752

[ref30] COMSOL Multiphysics, v. 5.6; COMSOL AB: Stockholm, Sweden, 1998.

[ref31] Lacy F. Developing a theoretical relationship between electrical resistivity, temperature, and film thickness for conductors. Nanoscale Res. Lett. 2011, 6, 636 10.1186/1556-276X-6-636. 22192792 PMC3284497

[ref32] aYang S.; Cho K.; Kim S. Energy devices generating and storing electricity from finger and solar thermal energy. Nano Energy 2020, 69, 104458 10.1016/j.nanoen.2020.104458.

[ref33] Hu J.; Zhang Y.; Cabero Z M. A.; Wei B.; Wei B.; Tu S.; Tu S.; Liu S.; Liu S.; Yu D.; Yu D.; Ansermet J.-P.; Ansermet J. P.; Granville S.; Granville S.; Yu H. Anomalous Nernst effect in Co2MnGa thin films with perpendicular magnetic anisotropy. J. Magn. Magn. Mater. 2020, 500, 166397 10.1016/j.jmmm.2020.166397.

[ref34] Jen S. U.; Yao Y.; Chen Y.; Wu J.; Lee C.; Tsai T.; Chang Y. Magnetic and electrical properties of amorphous CoFeB films. J. Appl. Phys. 2006, 99, 053701 10.1063/1.2174113.

[ref35] Uchida K.-i.; Kikkawa T.; Seki T.; Oyake T.; Shiomi J.; Qiu Z.; Takanashi K.; Saitoh E. Enhancement of anomalous Nernst effects in metallic multilayers free from proximity-induced magnetism. Phys. Rev. B 2015, 92, 094414 10.1103/PhysRevB.92.094414.

[ref36] Chen T.; Tomita T.; Minami S.; Fu M.; Koretsune T.; Kitatani M.; Muhammad I.; Nishio-Hamane D.; Ishii R.; Ishii F.; et al. Anomalous transport due to Weyl fermions in the chiral antiferromagnets Mn_3_X, X = Sn, Ge. Nat. Commun. 2021, 12, 572 10.1038/s41467-020-20838-1. 33495448 PMC7835387

[ref37] Guin S. N.; Vir P.; Zhang Y.; Kumar N.; Watzman S. J.; Fu C.; Liu E.; Manna K.; Schnelle W.; Gooth J.; et al. Zero-Field Nernst Effect in a Ferromagnetic Kagome-Lattice Weyl-Semimetal Co_3_Sn_2_S_2_. Adv. Mater. 2019, 31, 1806622 10.1002/adma.201806622. 31044469

[ref38] Chen T.; Minami S.; Sakai A.; Wang Y.; Feng Z.; Nomoto T.; Hirayama M.; Ishii R.; Koretsune T.; Arita R.; Nakatsuji S. Large anomalous Nernst effect and nodal plane in an iron-based kagome ferromagnet. Science advances 2022, 8, eabk1480 10.1126/sciadv.abk1480. 35030028 PMC8759748

[ref39] Shiomi Y.; Kanazawa N.; Shibata K.; Onose Y.; Tokura Y. Topological Nernst effect in a three-dimensional skyrmion-lattice phase. Phys. Rev. B 2013, 88, 064409 10.1103/PhysRevB.88.064409.

[ref40] Hanasaki N.; Sano K.; Onose Y.; Ohtsuka T.; Iguchi S.; Kézsmárki I.; Miyasaka S.; Onoda S.; Nagaosa N.; Tokura Y. Anomalous Nernst effects in pyrochlore molybdates with spin chirality. Phys. Rev. Lett. 2008, 100, 106601 10.1103/PhysRevLett.100.106601. 18352216

[ref41] Hasegawa K.; Mizuguchi M.; Sakuraba Y.; Kamada T.; Kojima T.; Kubota T.; Mizukami S.; Miyazaki T.; Takanashi K. Material dependence of anomalous Nernst effect in perpendicularly magnetized ordered-alloy thin films. Appl. Phys. Lett. 2015, 106, 252405 10.1063/1.4922901.

[ref42] Nagaosa N.; Sinova J.; Onoda S.; MacDonald A. H.; Ong N. P. Anomalous hall effect. Rev. Mod. Phys. 2010, 82, 1539 10.1103/RevModPhys.82.1539.

[ref43] Zhang F.; Wen F.; Lü Y.; Li W.; Lu Y.; Liu Z.; Xu B.; Yu D.; He J.; Tian Y. Proper scaling of the anomalous Hall effect in the Co/Pt multilayers. J. Appl. Phys. 2011, 110, 033921 10.1063/1.3622518.

[ref44] Canedy C. L.; Li X.; Xiao G. Large magnetic moment enhancement and extraordinary Hall effect in Co/Pt superlattices. Phys. Rev. B 2000, 62, 508 10.1103/PhysRevB.62.508.

[ref45] Lee W.-L.; Watauchi S.; Miller V.; Cava R.; Ong N. Anomalous Hall Heat Current and Nernst Effect in the CuCr_2_Se_4_– xBr_x_ Ferromagnet. Phys. Rev. Lett. 2004, 93, 226601 10.1103/PhysRevLett.93.226601. 15601108

[ref46] Zhang F.; Liu Z.; Wen F.; Liu Q.; Li X.; Ming X. Magnetoresistance and anomalous Hall effect with Pt spacer thickness in the spin-valve Co/Pt/[Co/Pt]_2_ multilayers. J. Supercond. Novel Magn. 2017, 30, 533–538. 10.1007/s10948-016-3820-8.

