# Supplementary material for: High-Power-Density Energy-Harvesting Devices Based on the Anomalous Nernst Effect of Co/Pt Magnetic Multilayers
Source: ACS Appl Energy Mater. 2022 Sep 9;5(9):11835–43. doi: 10.1021/acsaem.2c02422 (PMC9516660; doi:10.1021/acsaem.2c02422)
Supplement: Supplementary file 1 — ae2c02422_si_001.pdf [file ae2c02422_si_001.pdf]

Supporting Information

High-Power-Density Energy-Harvesting Devices Based on the Anomalous Nernst Effect of Co/Pt Magnetic Multilayers

Guillermo Lopez-Polin[,1], Hugo Aramberri[2], Jorge Marques-Marchan[1], Benjamin I. Weintrub[3], Kirill I. Bolotin[3], Jorge I. Cerda[1], Agustina Asenjo[*,1]*

[1]Instituto de Ciencia de Materiales de Madrid (ICMM-CSIC), 28049, Madrid, Spain

[2]Materials Research and Technology Department, Luxembourg Institute of Science and Technology (LIST), L-4362, Luxembourg

[3]Department of Physics, Freie University Berlin 14195 Berlin, Germany

AUTHOR INFORMATION

Corresponding Author

* Guillermo Lopez-Polin. e-mail: guillermo.lopez-polin@uam.es

*Agustina Asenjo: e-mail: aasenjo@icmm.csic.es

SI. 1 Device fabrication

a) *Macroscopic devices*

For the deposition of the macroscopic devices, we used Si/SiNx substrates of 25x20mm^2. We used a window of 15x15mm^2 to grow a multilayer of this size centred on the substrate. The deposition was performed by sputtering in a HV chamber with a base pressure of 10^{-6} mbar and deposition rates of ~0.1A/s. The heater and the heat sink were positioned in the top part of the substrates one at each side of the sample.

We prepared different multilayers with different thickness of Pt and Co. The hysteresis loops of the different multilayers have been measured by VSM (Vibrating-sample magnetometer) in two different configurations: applying perpendicular and parallel fields to the surface of the multilayer. Figure S1.1 shows the magnetic hysteresis curves of multilayers Co_x/Pt_y with x= 0.8nm, 0.5nm and 0.25nm and y= 1.7nm, 1.5nm, 1.1nm, and 0.8nm. Only the multilayers with 0.5nm of Co and 1.1nm, 1.5nm and 1.7 nm of Pt show a remanence in the perpendicular direction of ~100%.

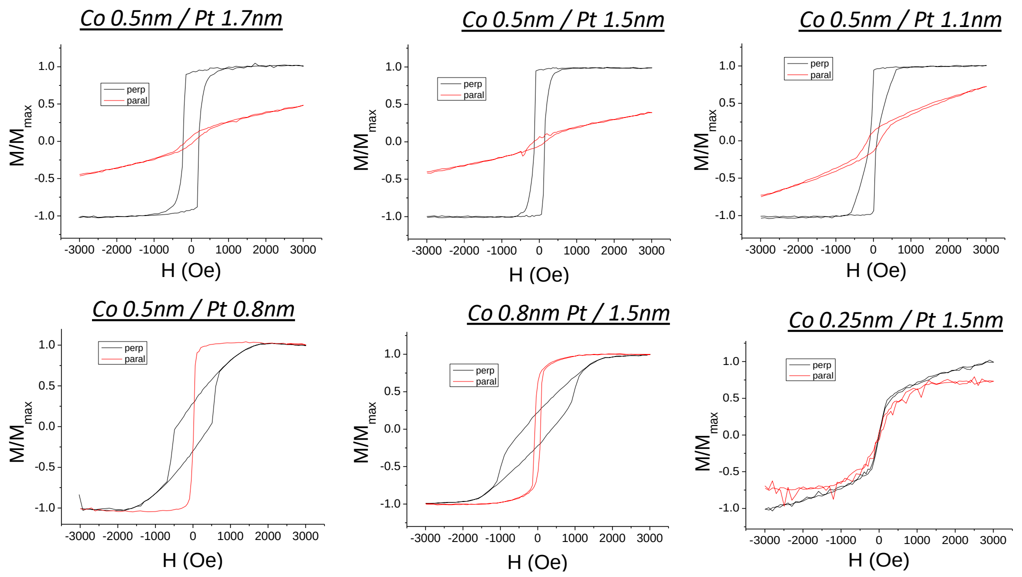

Figure S1.1 *Hysteresis cycles of several multilayers with different thickness of Co and Pt.*

We measured the ANE response of the samples by inducing an in-plane thermal gradient and measuring the voltage in the in-plane perpendicular direction while applying a magnetic field perpendicular to the surface of the multilayer of ~280mT (above the saturation value). Figure S1.2 shows the lineal trend of the ANE voltage as a function of the temperature gradient across the sample for different multilayers (left) and the dependence of the ANE coefficient with the thickness of Pt and Co (right).

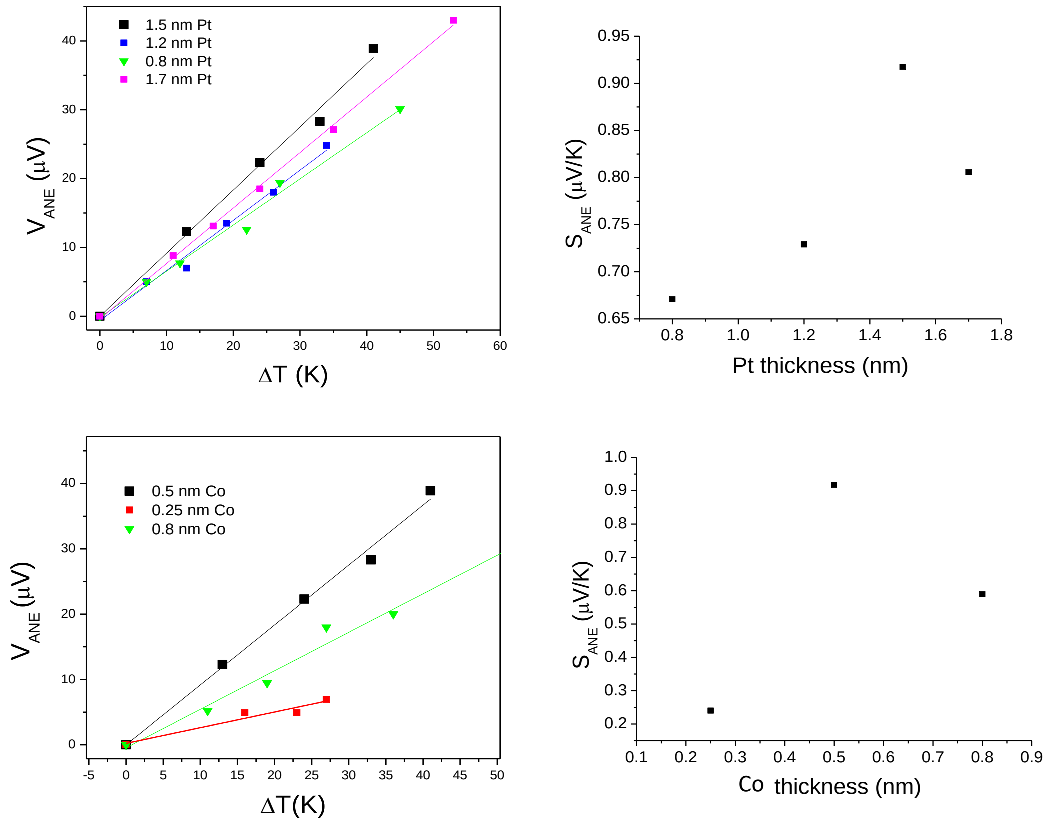

Figure S1.2 *Dependence of the voltage obtained as a function of the temperature gradient in the transversal direction for various multilayers with different Co and Pt thicknesses. The maximum ANE coefficient was found for the multilayer with 0.5nm of Co and 1.5nm of Pt.*

b) *Atomic structure of the multilayers as measured by TEM*

We performed TEM images of the multilayers in the "Servicio de Apoyo a la Investigación- *Servicio de microscopia electrónica de materiales*" in the university of Zaragoza. The goal was to obtain atomic resolution of the cross-section of the multilayer by TEM. To perform this experiment, it was necessary to obtain a lamella of the multilayer from the sample. To this end we deposited a layer of Pd on top of the surface to avoid electrostatic charges and we used a Focused Ion Beam (FIB) incorporated in a DUAL BEAM NOVA 200 system to cut a small piece with a thickness of ~25nm. The lamella was placed in an Image Corrected Titan TEM from FEI Company with a spherical aberration corrector (CEOS Company) at the objective lens. The images show the periodicity of the multilayers and also the atoms conforming the layers (figure S1.3). The roughness of the substrate induces a waving shape in the multilayer. We observed grains with uniform orientations of several nanometres size.

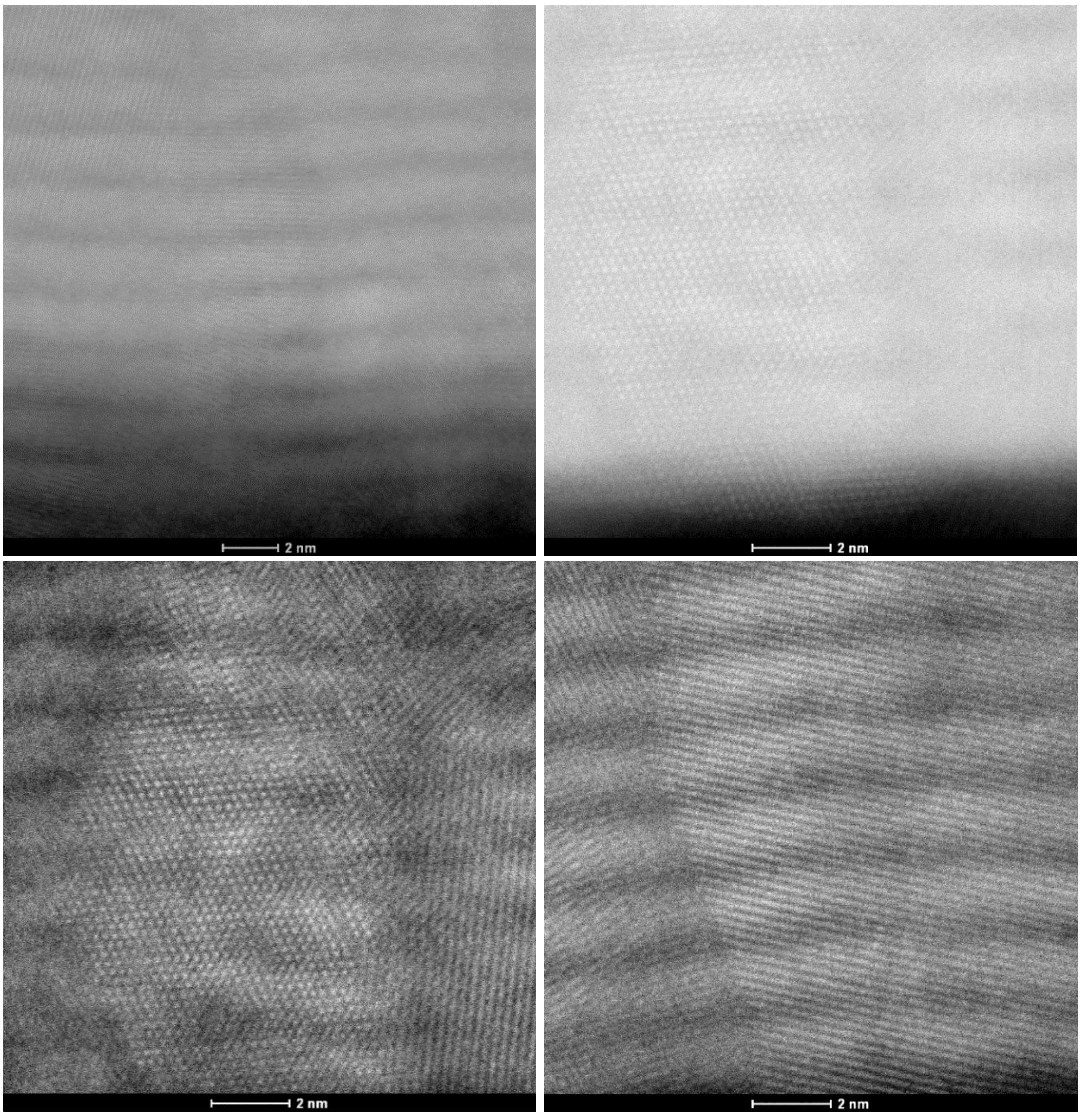

Figure S1.3 *TEM images of the* [$Co_{0.5nm}$/$Pt_{1.5nm}$]$_{10}$ multilayers.

c) Microscopic devices

Soda lime glass substrates, which have a poor thermal conductivity, have been used to prevent heat leakage out of plane. In addition, a thin layer of 50nm of alumina (Al_2O_3) was deposited on top of the glass to improve the in-plane thermal conductivity of the device. Rectangles of 100x5 μm^2 have been patterned by e-beam lithography to fabricate the multilayers bars. To increase the adhesion of the multilayers to the substrate, 1nm of Ti was thermally sublimated. After that, we deposited [Co/Pt]$_{10}$ multilayers by alternatively depositing 10 times 1.5 nm of Pt and 0.5nm of Co. The last layer of platinum was 2nm thick to better avoid oxidization of the cobalt. After that, we performed a second e-beam lithography for the heating and temperature sensing. Two 60nm thick Pt bars were deposited at a distance lower than 1µm, one at each side of the [Co/Pt]$_{10}$ rectangles. One of this bars acts as a heater and the other as a temperature sensor. For the temperature sensing, the resistance of the platinum bar was measured

by using the 4 probes technique to avoid the contact resistances. The measurements of the ANE was performed by controlling the setup with the WSxM software [1] through a Dulcinea from Nanotec Electronics.

SI. 2 COMSOL simulations

We performed finite element simulations using COMSOL Multiphysics[2]. COMSOL uses finite element modelling to compute the heat distribution. We used the Electromagnetic Heating interface, which includes the Electric Currents and Heat Transfer in Solids modules. The parameters for the materials used were given by the COMSOL library. Heat transfer coefficient of air (in contact with the top part of the device) was set to 5 W/(m^2·K), and the thermal conductivity of alumina 2.7 W/(m·K) [3]. The electrical conductivity ρ_e of Pt dependence of temperature T was obtained from the calibration using $\rho_e = R \cdot A/L$, where R is the resistance, A the cross section and L the length between the inner contacts of the Pt sensor. The thermal conductivity k of Pt was extracted from the electrical conductivity using the Wiedemann-Franz law $k \cdot \rho_e = L \cdot T$, where L is the Lorenz number and is set to $2.6 \cdot 10^{-8}\ \mathrm{W\Omega/deg^2}$ [4].

The thermal gradient in the *x* axis of the multilayer $\overline{\nabla T_x}$ and Pt probe temperature for different DC currents from 0 to 16 mA flowing through the microheater is evaluated. The geometry of the simulated device is shown in Figure S2, and the temperature distribution for a heater with a current I_{heater} of 10 mA (density current of 2.38·10^{10} A/m^2) is shown in figure S2.b. Figure S2.c represents the mean temperature of the Pt probe between the inner contacts T_{probe} as a function of the temperature gradient $\overline{\nabla T_x}$ averaged over the whole structure, which follows the equation $T_{probe}\ [\mathrm{K}] = (293.27 \pm 0.04)\ [\mathrm{K}] + (12.561 \pm 0.011)\ [\mathrm{\mu m}] \cdot \overline{\nabla T_x}\ [\mathrm{K/\mu m}]$. The temperature gradients $\overline{\nabla T_y}$ and $\overline{\nabla T_z}$, in *y* and *z* axes respectively, in the multilayer are considered negligible for the further analysis of ANE effect. Moreover, since the $\overline{\nabla T_y}$ is 1000 smaller than the $\overline{\nabla T_x}$ the influence of the Seebeck effect is our measurements is negligible.

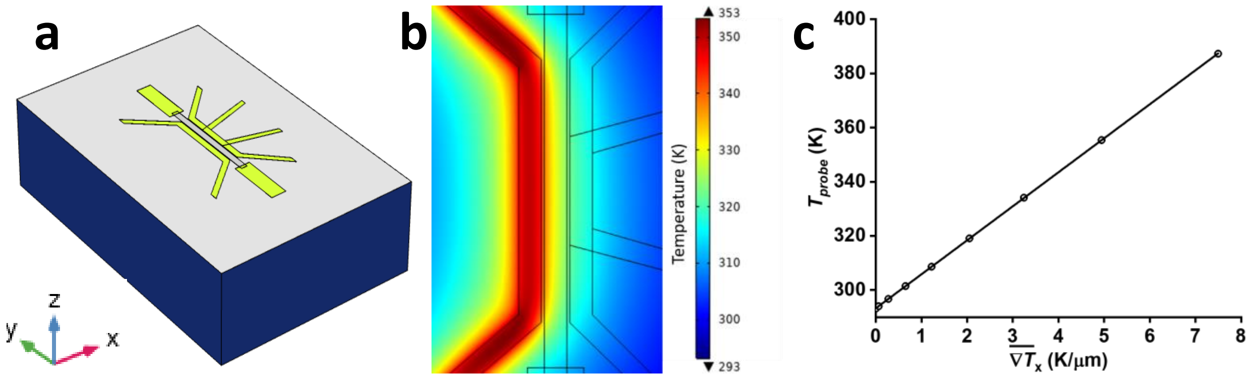

Figure S2 *a) Device geometry used for computing the temperature distribution. b) Thermal distribution for a DC current through the microheater I_{heater} of 10 mA. c) Simulated mean temperature of the Pt probe as a function of the thermal gradient in the structure along the x axis.*

SI. 3 Calibration of the dependence of the resistivity of the platinum bar with temperature

The devices consist of a Pt bar that act as a heater, the Co/Pt stripe and a third bar with 4 contacts that acts as a thermal sensor. The resistivity of Pt has a well-known linear dependence with temperature, of approximately 3850 ppm/K near ambient temperature, and is widely used as a thermal sensor. However, thin films are well-known to have different resistivity vs. temperature dependence than the bulk material [5]. Thus, we experimentally determine the dependence of the resistance of the Pt sensor with temperature. We simultaneously measure the resistance of the sensor and the temperature of the sample with a thermocouple positioned close (few mm) to the sensor while heating homogeneously the whole sample (figure S3). The experimental ratio of the increase of the resistance with the temperature is 2700 ppm/K, much lower than the value for bulk Pt .

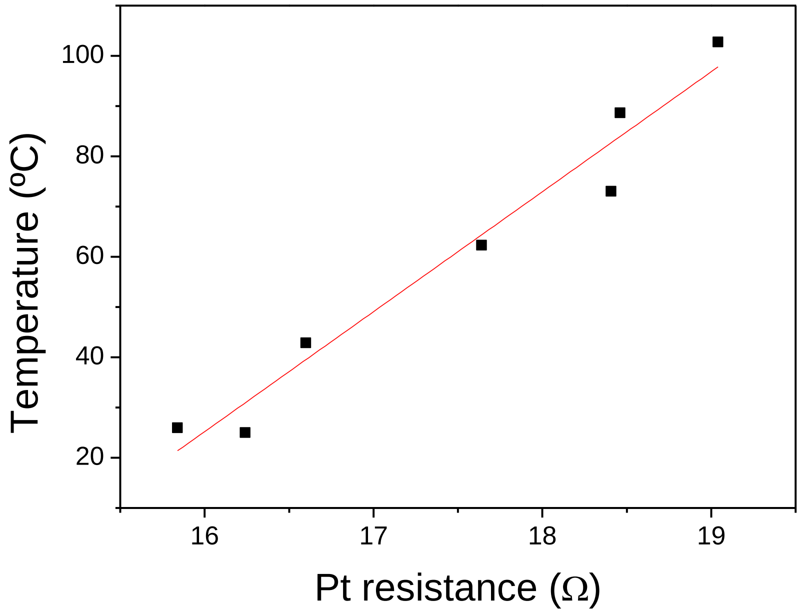

Figure S3 Resistance vs Temperature data of the Pt thermal sensor.

SI. 4 Maximum temperature gradient and ANE voltage in remanence

The remanence of the multilayers could be damaged when applying high temperatures. While at room temperature the structure is stable, when increasing the temperature domains in opposite direction appear in the multilayer and the magnetization of the sample tends to decrease. In our case, the maximum temperature gradient and ANE voltage that we could achieve without applying any field to the sample. The maximum Voltage was ~0.3mV and the maximum temperature difference across the multilayer was ~18K (~4K/µm).

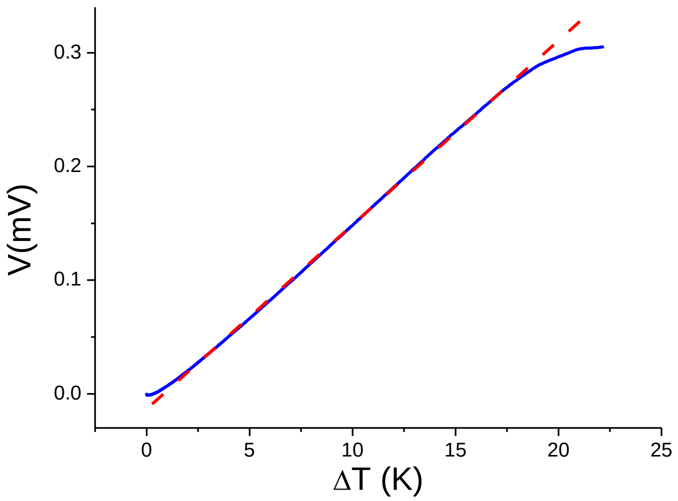

Figure S4 *V_{ANE} vs delta T curve obtained from a microscopic device of [$Co_{0.5nm}$/$Pt_{1.5nm}$]$_{10}$. At a thermal gradient of ~20K and a VANE of ~0.3mV the curve starts to deviate from the lineal tendency (dashed red line). We attribute the deviation to the appearance of domains in the opposite direction to the initial alignment due to thermal fluctuations. The deviation at low thermal gradients is due to the initial delay of the thermalisation of the system.*

SI. 5 V_{ANE} vs ΔT curves from experimental data

For the measurement of the Nernst effect of the microscopic devices we were able to directly determine 3 magnitudes: The voltage applied to the heating resistance, the resistance of the platinum thermal sensor and the transversal voltage generated due to ANE effect. From the resistance of the platinum bar we extracted the temperature of the sensor by calibrating the dependence of the resistance with temperature. From COMSOL simulations we related the average temperature of the sensor with the thermal gradient across the multilayer. At the end we were able to relate the thermal gradient across the Co/Pt structure with the resistance measured with 4 probe method.

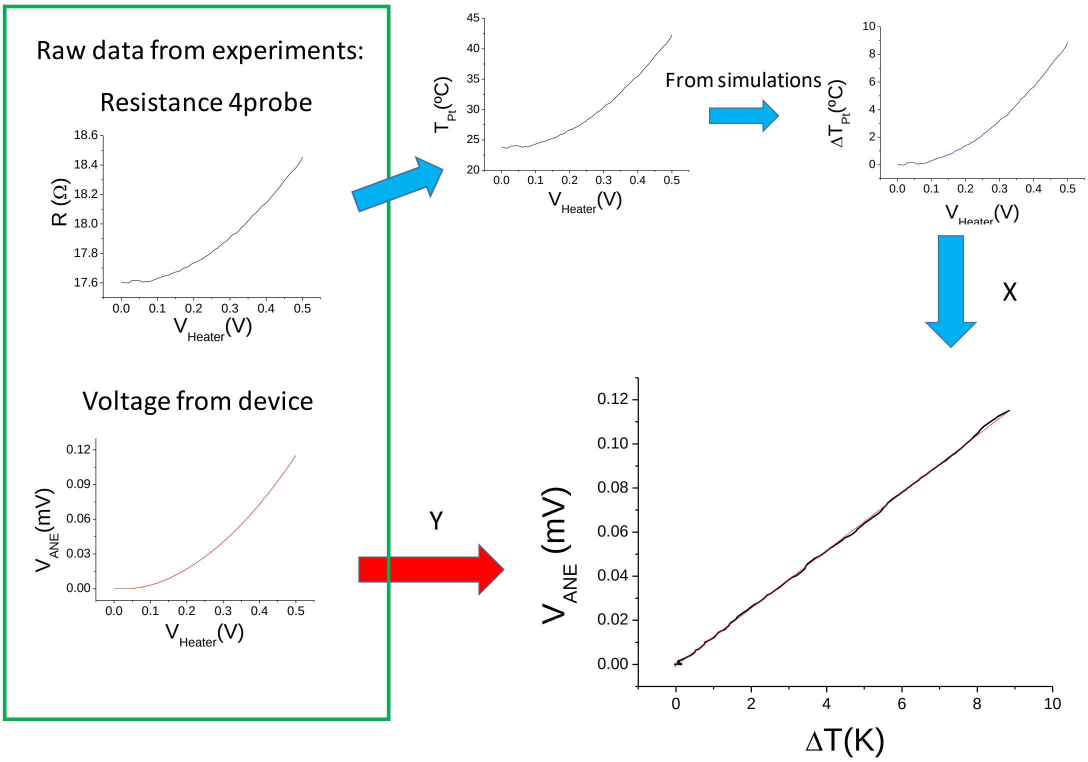

Figure S5 *Scheme of the method used to extract the V_{ANE} vs ΔT curves shown in the manuscript from the experiments.*

SI. 6 MFM images of the microscopic devices

The magnetization of the sample is obtained from the MFM images measured simultaneously with the ANE voltage (figure S6). From the maps we can quantify the parts of the multilayer with the magnetization pointing in both directions, and therefore, the total magnetization of the sample. We processed the images with WSxM software. The histogram of the MFM images exhibit two overlapping Gaussians, each corresponding to the domains pointing upwards and downwards. We counted the number of points at the two sides of the intersection of the two Gaussians, which gives the number of points pointing to each direction. The corresponding volume is calculated to obtain the magnetization of the sample at each magnetic field [6].

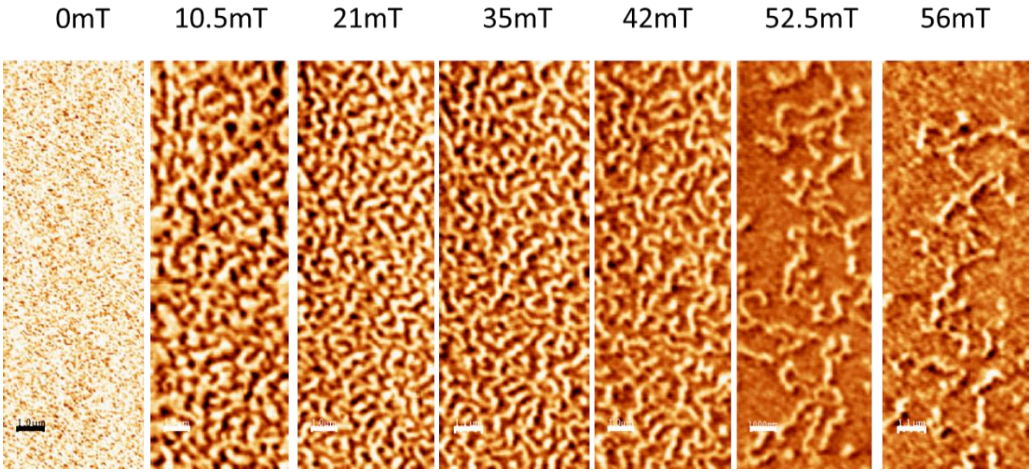

Figure S6 *MFM images of the [$Co_{0.5nm}$/$Pt_{1.5nm}$]$_{10}$ multilayers as a function of the positive applied magnetic field. The initial state corresponds to the remanence after applied a saturating negative magnetic field.*

In order to measure the magnetic domains of the multilayers while minimizing the influence of the magnetic moment of the tip on the sample, we prepare homemade low-moment tips. For that we deposited a thin film of Co onto the front face of commercial Nanosensors AFM tips (PPP-FMR). We found that tips with a 12.5nm-thick layer of Co were just above the lower limit of sensitivity of the AFM to measure the magnetic signal of Co/Pt multilayers. These tips are used to determine the magnetization of the sample from MFM images (figures 3.b and S6). Otherwise, for the rest of MFM images (figure 1.e), we use standard commercial Nanosensors MFM tips (PPP-MFMR).

SI. 7 First principles calculations

We obtain the atomic and electronic structure of the Co/Pt bilayers using first-principles density functional theory as implemented in the VASP package [7] [8]. To this end, we use the Perdew-Burke-Ernzerhof formulation of the generalized gradient approximation for the exchange-correlation functional [9]. We treat the atomic cores within the projector-augmented wave approach, considering the following states explicitly: 5*p*, 5*d* and 6*s* for Pt; 3*p*, 3*d*, 4*s* for Co. We set the plane-wave cut-off to 500 eV, for which the results converge well. We employ Monkhorst-Pack k-point grids [10] of 7x7x2 for the 2/2 and 3/2 bilayers, and of 7x7x1 for the 4/2 and 5/2 systems. Each atomic layer is taken as hexagonally compact. Within the Pt layer the stacking pattern is *fcc* (*abcabc*), while in the Co layer the *hcp* stacking pattern is followed (*ABAB*), and the Pt is assumed to take the hollow site on top of the Co layer.

We allow the structures to relax until the atomic forces became smaller than 1 meV/Å and the residual stress becomes smaller than 0.01 GPa. The spin-orbit coupling (SOC) is included self-consistently using the second-variation method, employing the scalar-relativistic eigenfunctions of the valence states [11] as implemented in VASP.

We project the DFT-derived wave functions onto Wannier functions using the Wannier90 package [12].

We then use the resulting Wannier-based models to compute the Nernst conductivity α_{xy} using the Mott relation as follows [13]:

$$\alpha_{xy} = \frac{e}{T\hbar}\sum_{n} \int \Omega_{ij}^{n}(k)\{f(\varepsilon_{nk})(\varepsilon_{nk} - \mu) + k_B T \, ln[1 + e^{-\beta(\varepsilon_{nk}-\mu)}]\}dk \qquad (3)$$

where e is the electron charge, T is the temperature, $\hbar$ is the reduced Planck's constant, Ω_{ij}^{n} is the Berry curvature of band n, f is the Fermi-Dirac distribution function, ε_{nk} is the band energy, μ is the chemical potential, k_B is Boltzmann's constant and $\beta = (k_B T)^{-1}$. To this end we implement the calculation of equation (3) within the Wannier Tools package [14]. The Brillouin zone integral in α_{xy} is performed over 200x200x67, 200x200x51, 200x200x42, and 200x200x36 k-point grids for the 2/2, 3/2, 4/2 and 5/2 heterostructures respectively, which we found to be well-converged.

The search for topological band crossings is done using the built-in function of Wannier. We use a very fine k-mesh (with points every $7\cdot10^{-4}$ $Å^{-1}$) looking for band energy differences of less than 1 meV in the Wannier models for the heterostructures with SOC.

The results are shown in Figure S7. We see that thermoelectric conductivity oscillates with energy with an amplitude of a few $Am^{-1}K^{-1}$, peaking close to at E=+0.4 eV. However, at the computed Fermi energy the absolute values are not particularly large (except perhaps that of the 2/2 heterostructure). More importantly, α_{xy} at the Fermi level changes sign with the addition of each Pt monolayer, which is at odds with our experimental measurements. Even considering that the computed Fermi levels could be slightly off, in order to attribute a (dominant) topological origin of the ANE effect in the Co/Pt heterostructures there should be an energy window in which the computed thermopower does not change sign with Pt thickness and where the changes in the thermopower are not very large among the studied systems (the measured changes in ANE with Pt thickness are quite modest, of the order of 30 %, see SI2). This does not seem to be the case in the vicinity of the Fermi level, and could only be justified if the Fermi level were at +0.4 eV, but such a large error in the Fermi energy seems unlikely.

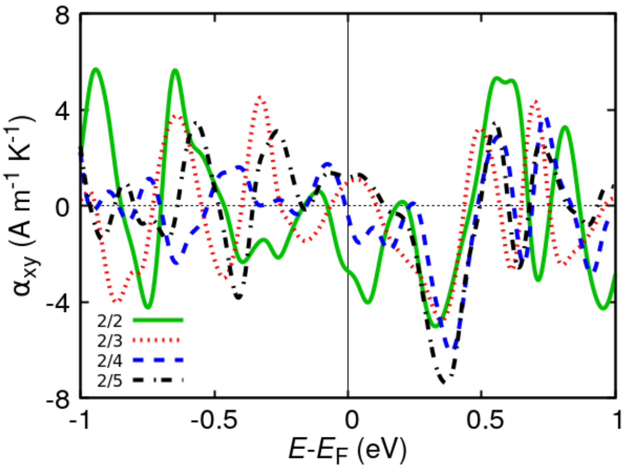

Figure S7 *Room temperature thermoelectric coefficient* α_{xy} *as a function energy as computed from first principles for Co/Pt heterostructures. The results in solid green, dotted red, dashed blue and dot-dashed black correspond to a Pt thickness of 2, 3, 4 and 5 monolayers, respectively (see also legend). The Co is fixed to 2 monolayers in all cases.*

SI. 8 Anomalous Hall effect measurements.

In order to extract information about the extrinsic or intrinsic origin of the ANE, we perform AHE measurements of the Co/Pt multilayers. To this end we grow a Co0.5nmPt1.5nm multilayer with H shape. We apply a current through the bar and measure the voltage between two of the contacts at the same side to determine the longitudinal resistivity (ρ_{XX}) and two contacts in opposite sides to determine the Hall resistivity (ρ_{XY}) of the multilayer.

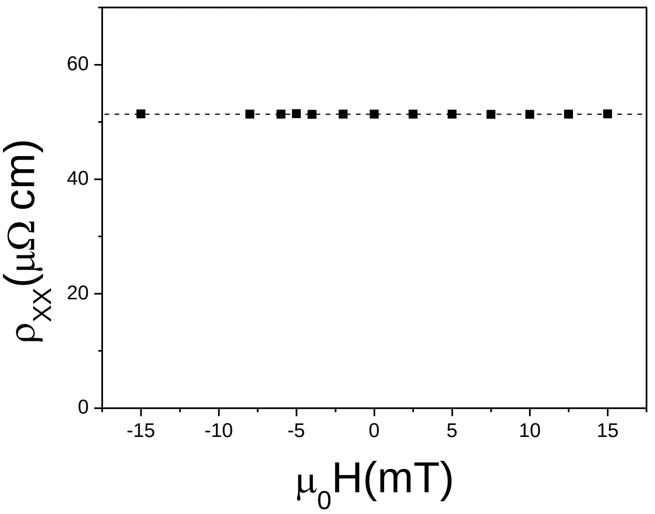

Figure S8 *Longitudinal resistivity as a function of the magnetic field. The resistivity is constant with the field, showing no magnetoresistance. The dashed red line is place at a constant value of* ρ_{XX} *to guide the eye.*

SI. 9 Sensitivity of macroscopic devices

In order to test the sensitivity of the ANE voltage generated in the magnetic multilayers to the thermal gradient, we measured the voltage generated due to thermal energy caused by the human body by pressing with the finger one of the sides of the macroscopic $[Co_{0.5nm}/Pt_{1.5nm}]_{10}$ multilayer in remanence. Figure S8 shows the ANE voltage obtained from the device as a function of the time. During this time, we pressed and lift up three times the finger. The voltage due to the small temperature difference caused by the finger was small but measurable, of about 2μV.

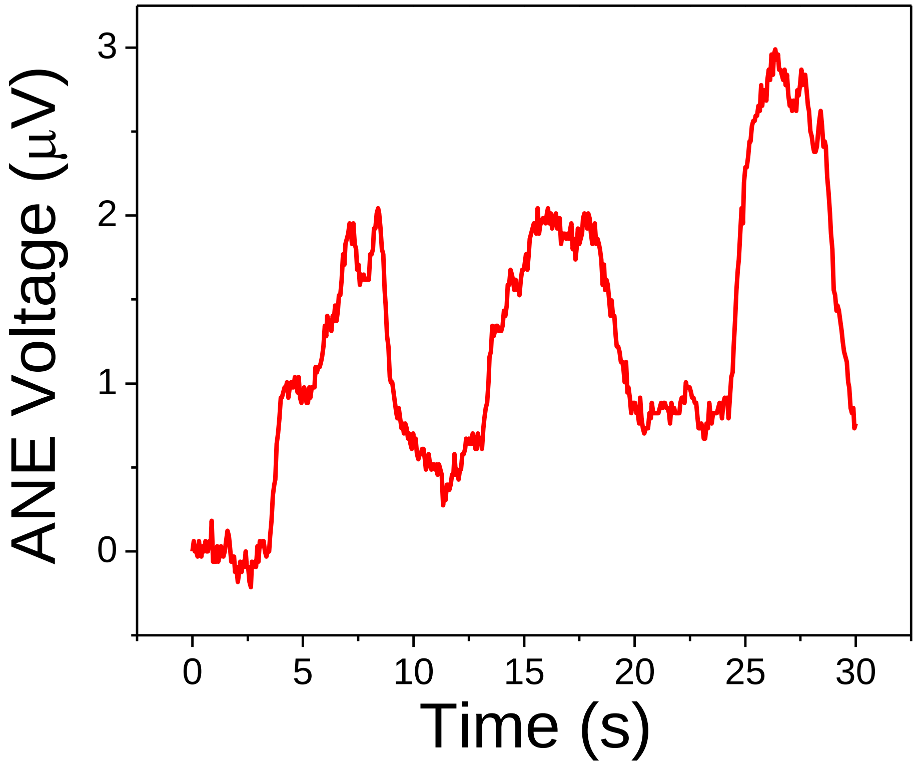

Figure S9 *ANE voltage obtained when pressing with the finger one of the sides of the samples.*

(1) Horcas, I.; Fernández, R.; Gomez-Rodriguez, J.; Colchero, J.; Gómez-Herrero, J.; Baro, A. WSXM: a software for scanning probe microscopy and a tool for nanotechnology. *Review of scientific instruments* **2007**, *78* (1), 013705.

(2) *COMSOL Multiphysics® v. 5.6, www.comsol.com*; COMSOL AB: Stockholm, Sweden, (accessed.

(3) Cappella, A.; Battaglia, J. L.; Schick, V.; Kusiak, A.; Lamperti, A.; Wiemer, C.; Hay, B. High Temperature Thermal Conductivity of Amorphous Al_2O_3 Thin Films Grown by Low Temperature ALD. *Advanced Engineering Materials* **2013**, *15* (11), 1046-1050.

(4) Kittel, C. Introduction to solid state physics, John Wiley & Sons. *Inc., Sixth edition,(New York, 1986)* **2005**.
(5) Lacy, F. Developing a theoretical relationship between electrical resistivity, temperature, and film thickness for conductors. *Nanoscale research letters* **2011**, *6* (1), 1-14.
(6) Asenjo, A.; Jaafar, M.; Navas, D.; Vázquez, M. Quantitative magnetic force microscopy analysis of the magnetization process in nanowire arrays. *Journal of applied physics* **2006**, *100* (2), 023909.
(7) Kresse, G.; Hafner, J. Ab initio molecular dynamics for open-shell transition metals. *Physical Review B* **1993**, *48* (17), 13115.
(8) Kresse, G.; Furthmüller, J. Efficiency of ab-initio total energy calculations for metals and semiconductors using a plane-wave basis set. *Computational materials science* **1996**, *6* (1), 15-50.
(9) Perdew, J. P.; Burke, K.; Ernzerhof, M. Generalized gradient approximation made simple. *Physical review letters* **1996**, *77* (18), 3865.
(10) Chadi, D. Special points for Brillouin-zone integrations. *Physical Review B* **1977**, *16* (4), 1746.
(11) Koelling, D.; Harmon, B. A technique for relativistic spin-polarised calculations. *Journal of Physics C: Solid State Physics* **1977**, *10* (16), 3107.
(12) Mostofi, A. A.; Yates, J. R.; Lee, Y.-S.; Souza, I.; Vanderbilt, D.; Marzari, N. wannier90: A tool for obtaining maximally-localised Wannier functions. *Computer physics communications* **2008**, *178* (9), 685-699.
(13) Xiao, D.; Yao, Y.; Fang, Z.; Niu, Q. Berry-phase effect in anomalous thermoelectric transport. *Physical review letters* **2006**, *97* (2), 026603.
(14) Wu, Q.; Zhang, S.; Song, H.-F.; Troyer, M.; Soluyanov, A. A. WannierTools: An open-source software package for novel topological materials. *Computer Physics Communications* **2018**, *224*, 405-416.
